# PACE Labels on Healthy and Unhealthy Snack Products in a Laboratory Shopping Setting: Perception, Visual Attention, and Product Choice

**DOI:** 10.3390/foods10040904

**Published:** 2021-04-20

**Authors:** Clara Mehlhose, Daniel Schmitt, Antje Risius

**Affiliations:** Marketing of Agricultural and Food Products, Department of Agricultural Economics and Rural Development, University of Göttingen, Platz der Göttinger Sieben 5, 37073 Göttingen, Germany; daniel.schmitt@sgs.com (D.S.); a.risius@uni-goettingen.de (A.R.)

**Keywords:** eye-tracking, consumer behavior, calorie information, nutritional labeling, food-choice

## Abstract

Informative food labels are one way to increase nutritional awareness in society and can essentially help individuals maintain balanced dietary practices. Nonetheless, making food labels ‘informative’, in the sense of applicability, is not always easy. Physical activity calorie equivalent (PACE) food labeling is one approach to achieve this goal. Yet, it is neither understood how consumers perceive PACE labels, nor how effective they are in regards to healthy food choices. Moreover, it is of interest to assess the perception of real products in close-to-realistic environments. Therefore, this study examined a simulated purchase situation and consumers’ visual attention on PACE labels—on 20 different real snack products with varying health values. In a laboratory-shopping environment, the gaze behaviors of 91 consumers were examined with a head-mounted eye-tracker. In regards to perception, it was elucidated that every participant noticed at least one PACE label. On average 1.39 PACE label fixations on different products were counted with a mean fixation duration of 0.55 s and a mean time to first fixation of 22.46 s. On average, 22.9% of the participants viewed the PACE labels at least once, but the intensity and duration varied greatly between the different products; ’healthier products’ attracted more visual attention than ‘unhealthier products’. In regards to health choice, it became obvious that the choices observed were rather healthy and PACE labels attracted attention. This may have been especially true for participants with little involvement in physical activity and health behavior, which may have been the main target group. Hence, catchy, communicable PACE labels, as well as balanced product offerings may facilitate more healthy food choices. The real-world laboratory setting offered valuable insights, which should be followed-up on.

## 1. Introduction

The global rising obesity rate (of almost 40%) will seriously burden national health systems [1,2]. To face obesity and its associated secondary diseases, keeping a low-energy, micronutrient-rich diet, combined with sufficient physical exercise is one of the most important aims, especially in industrialized countries [3]. Due to changes in living and working conditions, individual habits, low levels of nutritional knowledge, and the constant availability of food and food cues in our society, maintaining this balanced diet currently poses as a challenge for many people [4,5]. One way to increase nutritional awareness and encourage healthier eating decisions is nutritional labeling, which is mandatory in many countries; for example, in European countries, the majority of pre-packed foods must have a nutrition declaration. However, it is known that the current front of pack (FoP) nutrition information on foods and beverages is limited in its effectiveness, because many consumers have difficulty processing the provided information [6,7]. This highlights the need for more easily understandable caloric information, provided in a more intuitive, yet action-oriented way, e.g., in the form of physical activity calorie equivalent (PACE) food labeling.

The idea of PACE labeling is to provide a translation to abstract to interpret product information, such as the calorie content information, into intuitive units. It interprets them as an equivalent to the number of calories contained in the specific product, e.g., as in this case in minutes to walk or to run. This is expected to simplify the processing of label information, as well as orienting it into an action-based result, because lower levels of nutritional and numerical competence are required [8,9].

Labeling intervention strategies aim to disrupt habitual food decision situations at the point of purchase and, therefore, focus on quickly catching consumers’ attention by providing information that does not require deep cognitive processing [10]. In this context, PACE labeling schemes seem particularly promising, because a combination of a colorful graphic with information about the content enables easy understanding, which is expected to shorten the processing time of the provided information [11,12]. This, again, may result in a greater influence of point-of-purchase labeling on consumer behavior towards healthier eating choices [7,9,13,14,15,16]. Further, this approach might promote physical activity [17].

To the best of our knowledge, PACE labels are not yet in use, but the Royal Society for Public Health in the UK called for an introduction of PACE labeling in 2016 [18]. Until now, several studies and meta-analyses have shown effects of PACE labels in reducing calorie consumption and improving food choices. This especially describes the effects of the labels in comparison to the absence of food labels. Overall, PACE labels may have been effective at reducing the number of kilocalories of food consumed, but compared to calorie-only labeling, they did not reduce calorie consumption [9,15,19,20,21].

From a consumer point of view, the perception of PACE labels has not been sufficiently investigated. It is very likely that consumer perceptions of PACE labels are very different depending on the presentation of the experimental stimuli (product pictures vs. real products and single product vs. product category) [15]. Most evidence from previous studies were from laboratory settings or hypothetical meal selection scenarios, and there has been a lack of studies that applied PACE labels in real-world settings or used real products to examine the perception of labels. This seems particularly critical against the background that PACE labels are discussed worldwide as an alternative labeling approach [22]. Therefore, in this study, we examined PACE labels that were realistically attached to various real snack products with differing health values, and presented in a laboratory-shopping shelf. The purpose was to obtain a better understanding of consumer perceptions of PACE labels, on real products, and the attention they gained in a simulated purchase situation. As traditional consumer research techniques allow only the measurement of conscious reactions to stimuli, we applied, in addition to a survey, a more indirect consumer neuroscience approach—eye-tracking technology. This enabled us to measure immediate, more unconsciously aroused physiological reactions to the PACE label stimuli. Hence, the overall hypothesis is that PACE labels need visual framing in order to be effective in calorie reductions. In order to come closer to test this, we designed the study to, at first, test the effectiveness of the visual stimuli.

In this paper, we investigated the visual attention of PACE labels, and whether they were fixated more intensely (in the sense of longer, faster, and more often) compared to other product information using eye-tracking technology (ET). Other product information, in our case, included the price of the product and the product as a whole. Further, we investigated if PACE labeling was effective in regards to product choices with different health values. Moreover, we investigated whether (and how) the participants perceived and remembered the PACE labels during purchasing. For this, we used a questionnaire and contrasted the results with the ET measurements.

## 2. Materials and Methods

### 2.1. Eye-Tracking Methodology

There is a need to measure the effects of nutritional labeling, not only with stated preferences or other retrospective methods, which are limited (i.e., in regards to the relevance and external validity of their findings), but also with actual behavioral measurements [23]. To examine the basis of consumer behavioral patterns, concerning visual attention and perception of PACE labels on real products, this study used a head-mounted Tobii Pro Glasses 2 system. This system gains information at a sampling rate of 50 Hz and consists of a binocular corneal reflection and dark pupil tracking to guarantee an absolute pupil measurement during the recording time. The head unit of the glasses consists of four eye cameras and one wide-angle HD-scene camera at the front. It has a resolution of 1920 × 1080 pixels and a frame rate of 25 frames per second. The front camera records at a maximum angle of 82° horizontally and 52° vertically. The system is mounted as regular glasses, which enables natural viewing behavior in real-world environments. In this study, we were interested in the detailed visual information processing of the PACE labels on different products, which is why we focused on participant fixations. Furthermore, the PACE labels can be considered bottom-up factors, as we expect them to attract the attention of consumers. Since the labels are not available on the market, they are unknown to consumers. As the participants were not given direct/specific search orders, but were asked to pursue a snack-choice in a buying situation, it can be assumed that various top-down factors would direct their visual attentions. For example, the search for products that are healthy, for a specific brand or a drink, instead of a snack alongside the applied PACE label information.

### 2.2. Participants

A total of 102 subjects were recruited to take part in this study. The eye-tracking experiment took place at a German university in February 2019. The study was conducted in accordance with the Declaration of Helsinki and with the principles of the ICC/ESOMAR Code. Our study did not impose unreasonable stress to the participants nor did it harm their bodily or psychological well-beings. All participants provided written informed consent to take part and were informed about the opportunity to withdraw from the study at any point in time. They received a monetary compensation for the time allowance (around 20 minutes/assessment) at the end of the study (EUR 5.00). Before the experiment started, all participants received instructions about the testing procedure, had the possibility to ask questions, and were informed that they could withdraw from the study at any time without consequences. A total of 11 participants had to be excluded due to poor data quality of the eye-tracking measurement (cut-off value < 80% data quality). Therefore, data analysis was performed with 91 recordings. A total of 52 participants were female, and 39 were male. The mean age was 26.4 years (ranging from 18 to 40 years), and the average body mass index (BMI) was 22.58, ranging from a minimum of 16.8 to a maximum of 32.6.

### 2.3. Stimuli and Experimental Setup

**Figure 1:**
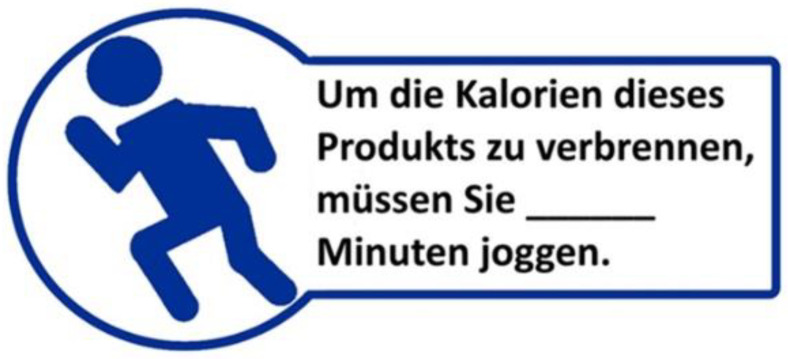
Design of the physical activity calorie equivalent (PACE) label used in the eye-tracking (ET) experiment. The reference text on the claims translated into English reads as follows: “To burn the calories of this product, you have to run for … minutes”.

The PACE label consisted of a blue symbol of a person running, with accompanying information on the physical activity calorie equivalent. We used a circular symbol of a person running, combined with a rectangular text field. It indicated the time it would take to complete a physical activity (in this case, jogging) to burn the equivalent number of calories of the particular product. The reference text on the claims translated into English reads as follows: “To burn the calories of this product, you have to run for … minutes.” The color of the symbol, as well as the label frame, was blue, because we focused on a more neutral color than green and red, as these are often associated with health/organic (green) or ban/warning (red) [24]. The label was designed purely for this study and was added to all products of the supermarket store, by a specific label-sticker. To depict a real-life scenario, with close to real-life label sizes, we chose the size of the PACE labels according to the size of the product packages, and tried to make them appear as realistic as they would in real-life. The labels measured 4.5 × 1.5 cm (respectively 2 cm at the highest point). An exemplary illustration of the PACE label is shown in Figure 1.

The PACE labels were printed and applied by the means of small stickers, and all of them were then attached onto a variety of 20 different snacks and soft drinks. All products were labeled with their respective PACE label based on the number of calories contained in the product. We used an online calorie tracker to convert the number of calories into physical activity and calculated the label values under the assumption that a person with a body weight of 70 kg burned 280 kcals when jogging slowly for 30 min (see also www.fitforfun.de (accessed on 16 April 2021), caloric calculator). We then rounded the exercise values (declared on the label) up or down so that they were shown in five-minute increments on the labels. The values ranged from 0 min to a maximum value of 60 min. All products were purchased at a German supermarket and, hence, were already available; there were no hypothetical products. The product selection was based on a thorough discussion, and pretesting within the research group of eight students and two principal investigators in charge. When selecting the products for this study, care was taken to create the widest possible range of popular soft drinks and snacks with different perceived health values. Market-leading manufacturers and a selection of organic products were included. Further, products with comparable calorie values, but different associated health values (‘healthy’ alternatives, such as fruit bars; ‘unhealthy’ alternatives, such as a chocolate bars) were selected. These included organic products, fruits, trail mixes, and drinks. By sorting the complex health values we argued from a consumption perspective and balanced the healthy vs. unhealthy options (inspired by systematic design of choice experiments) to designate two groups with comparable mean number of minutes: mate tea, orange juice, water, banana, apple chips, oat biscuits, oat bar, fruit bar, trail mix, and peanuts in one group (= healthy snacks, mean: 27.5 min). The other group contained: Coke, Coke sugar-free, energy drink, fruit juice drink, nut–nougat–crème snack, chocolate muesli bar, chocolate bar, chocolate biscuits, chocolate, and potato chips (= unhealthy snacks, mean: 26 min). Table 1 shows an overview of all selected products, their number of calories (per 100 g or mL and per product), the physical activity equivalent values in jogging units (per min), as well as the corresponding product prices.

**Figure 2:**
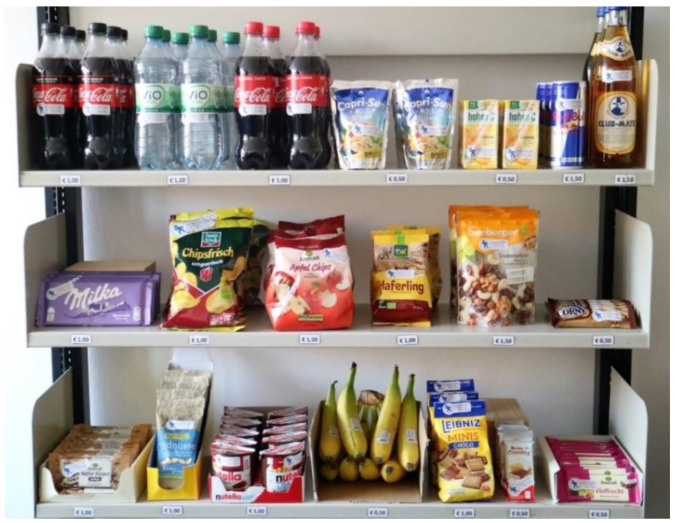
Setup of the snack shelf in the laboratory supermarket during the eye-tracking experiment.

To simulate a supermarket situation, all products were placed on a shelf as in a real shopping situation (see Figure 2). All products were arranged in multiple rows, so that it looked similar to a supermarket shelf. Since it was aimed to simulate a real purchase situation, price information was attached to the front of the shelves. The prices ranged from EUR 0.50 to EUR 1.50 in increments of EUR 0.50 and were within the typical price range for these product categories in Germany, which had previously been validated through an inventory in different shops. The prices had been rounded up/down to simplify the purchase process. The shelf, the positioning of the products, as well as the pricing information, are shown in Figure 2.

### 2.4. Study Procedure

The experiment took place in a laboratory room at a university located in Germany. It contained no furniture or wall decorations other than a table and chair for the subject, as well as the prepared product shelf that including 20 different snacks and soft drinks, and a black board for the calibration of the eye-tracker. To ensure constant lighting conditions, the window front was shielded. After providing general instructions about the experimental setting, the test persons were informed about the methodical processes and the test procedure. Then, the eye-tracking glasses were given to the participant, with instructions to wear them like normal glasses. The use of the eye-tracker was not affected by the presence of a visual aid and could simply be placed over normal glasses. To calibrate the eye-tracking glasses, participants had to look at the black billboard, where a calibration template was attached to the middle of the black background. Due to the head-mounted glasses used in this study, the participants were not restricted in their movements, which enabled them to behave naturally when looking at the shelves, when taking the products off to read the information on the back, and so on.

Participants received the following instructions concerning their task: “We have set up a small test supermarket for you. Take your time and look at the products. If a snack appeals to you, please select it. After the experiment is completely finished, you will receive the monetary compensation and pay for the snack at the checkout. Your selection is binding. Please start shopping in our supermarket now.” Participants were not forced to select and buy products. A choice was made when they grabbed the product(s) and informed the test supervisor that they finished their purchase. There was no limit in terms of the number of products to purchase. After the participants had chosen a product, the recording was stopped, and the eye-tracking glasses were removed. Immediately following the end of the ET experiment, the participants were asked to fill in a questionnaire, which contained general questions about the food, sleep, and movement behaviors of participants. In it, the first three questions were open questions, aimed to discover whether the participants recognized the PACE labels, and what information they were looking for on the products. In the end, the chosen product was paid; participants received monetary incentive and could keep the chosen product(s).

### 2.5. Data Analysis

**Figure 3:**
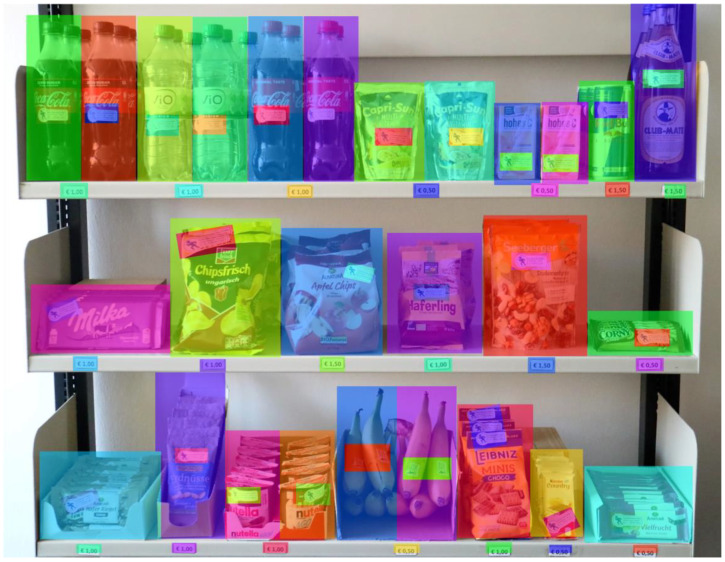
Static AOIs covering the PACE on every product and the price signs in front of the products.

The analyses of eye-tracking data were performed with the software Tobii Pro Lab x64 (version 1.111.19220; Tobii Technology, Stockholm, Sweden). The datum used to analyze was a picture taken of the shopping shelf, as shown in Figure 2. Static area of interest (AOI) was defined for each product as a whole, another AOI covered the PACE label on the specific product. In addition, each price label in front of the product received an AOI (for the AOIs, see Figure 3). The label and price AOIs were almost consistent, in terms of size and shape. Due to the different sizes and shapes of the real products and the resulting optical differences on the photo used as a snapshot, there were smaller deviations (in millimeters) in the size of the AOIs. It is assumed that these deviations hardly influenced the trends in label perceptions.

Three eye-tracking metrics were used to analyze the visual attention on the AOIs: *Time to first fixation* (TTF): this is the time period from the onset of the stimuli to the first fixation of a specific AOI (in seconds). *Fixation count* (FC): this metric counts the number of fixations that the participant makes to one specific AOI during the recording. *Total fixation duration* (TFD): this describes the length of each single fixation within the AOI (in seconds). Univariate methods were used to show descriptive statistics about the participants and their gaze behaviors. This included mean gaze durations and proportions of participants gazing at an AOI. Single factor analysis of variance (ANOVA) was used to calculate differences between different groups of mean values, e.g., whether the mean values of the labels differed significantly from those of the prices and the products, in regards to TFD, FC, and TTF. All statistical analyses were performed with SPSS (IBM, SPSS Statistics 26) and MS Excel 2010. The evaluation of the open questions from the questionnaire was based on qualitative content analysis according to Mayring [25].

## 3. Results

### 3.1. Sample Description

Table 2 describes the sociodemographic characteristics of the sample (*N* = 91). It must be mentioned that the participants were recruited at a university, which means that they were, for the most part, students and, therefore, not representative of the German population. The gender distribution in the survey showed a slightly higher proportion of women (sample: 57.1%, German population: 51.2% female) [26]. The average age of 26.4 years of the participants was about 18 years below the German average of 44.4 years [26]. Furthermore, 33.3% of the participants already had a university degree (German population: 15.6%), and it can be assumed that the majority were still in university. The population group enrolled in higher education was, therefore, clearly overrepresented in the present sample. Concerning the physical activity of the participants, the results showed that most participants recorded pursuing low- or medium-intense physical activity. More than 60% of participants performed low-intensity physical activity more than two hours a week. More than 60% of participants were active for more than one to two hours, or more than two hours a week (medium physical activity). More than half of the participants performed physical activity more than one to two hours, or more than two hours per week (high intensity), but almost 18% declared not to perform high intensity physical activity ever. The number of participants who recorded not performing physical activity at all, or less than one hour per week, was less than 10%.

### 3.2. Eye-Tracking Data

#### 3.2.1. Heatmap

**Figure 4:**
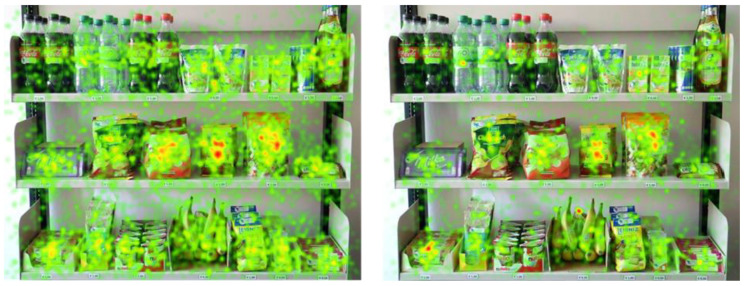
Heatmap of absolute fixation counts and absolute fixation duration.

Figure 4 shows the absolute fixation duration and absolute fixation count of the snack shelf in a heatmap to provide a general overview and to visualize the view of the shelf as a whole. In our case, items that were viewed longer and more intensely compared to the other products were apple chips, oat biscuits, trail mix, and oat bars.

#### 3.2.2. Metrics

Overall, the average total time of interest duration, which is the length of time that the participants concentrated on the shelf, was 43.05 s (SD: 23.45 s). The average total recording duration was 71.07 s (SD: 34.37 s). Moreover, 79% of the participants viewed the AOIs of the products at least once for a total mean view time of 24.27 s. Moreover, 22.91% viewed the PACE label AOIs at least once for a total mean view time of 3.51 s. For the price signs, this holds true for 5.93% of the participants, with a total mean view time of 0.41 s.

#### 3.2.3. Time to First Fixation

In general, the products had 71.88 fixations, whereas oat biscuits (89 times), trail mix (86 times), and apple chips (85 times) had the highest number of counts. The labels had on average 20.85 fixations, with oat biscuits (42 times) having the highest number of counts, followed by trail mix (34 times) and banana 1 (31 times). The prices had 5.33 fixations on average, with chocolate (10 times) and chocolate muesli bar and oat biscuits (each nine times) having the highest counts. The products were, on average, first fixated after 12 s, the price signs after 22.39 s, and the labels after 22.47 s. The products with the shortest time to first fixation were fruit juice drink 1 (5.82 s), apple chips (7.04 s), and trail mix (8.33 s). The labels that were fixated fastest were fruit juice drink 1 (10.17 s), Coke 2 (11.35 s), and energy drink (13.72 s). For the price signs, fruit juice drink (6.65 s), energy drink (9.11 s), and medium water (10.95 s) had the shortest time to first fixation. For details, see Table A1. The products as a whole were fixated significantly faster than the labels (*p* ≤ 0.001) and the prices (*p* ≤ 0.001); the labels and the prices did not differ significantly. A comparison among the AOIs of the product, label, and price, in relation to the different health value product groups, showed that the TFF did not differ between the healthy and unhealthy product alternatives, nor between their labels and price signs. However, a more detailed comparison between the price signs of the products with differing health values but with a comparable number of minutes (healthy products–low minutes vs. unhealthy products–low minutes, and healthy products–high minutes vs. unhealthy products–high minutes) showed that the price signs of the unhealthy–high minute products were observed significantly faster compared to the healthy–high minute products (*p* ≤ 0.05).

#### 3.2.4. Fixation Counts

The products that had the highest number of participants with at least one fixation on the AOI were oat biscuits (97.8%), trail mix (94.5%), and apple chips (93.4%). For the labels, this held true for oat biscuits (46.15%), trail mix (37.36%), and banana 1 (34.07%), for the price signs for chocolate (10.99%), followed by oat biscuits and the chocolate muesli bar (both 9.89%). On average, the products were fixated 3.3 times per participant, the labels 1.39, and the price signs 1.1 times. For the products, trail mix (6.66), apple chips (5.54), and oat biscuits (5.1) had the highest number of fixations. For the labels, trail mix (1.88), energy drink (1.81), and chocolate bar (1.8), and for the price signs, apple chips (1.5), chocolate (1.3), and chocolate bar (1.25) had the highest number of fixations. For details, see Table A2. The products had a significantly higher number of fixations compared to the labels (*p* ≤ 0.001) and compared to the prices (*p* ≤ 0.001); further, the labels had a significantly higher number of fixations compared to the price signs (*p* ≤ 0.001). When it came to the comparison of the different health value product groups, the AOIs of the healthier products had a significantly higher number of fixations (*p* ≤ 0.05) compared to the unhealthy product group. There were no significant differences between the price signs and labels of these two groups.

#### 3.2.5. Total Fixation Duration

The average total fixation duration for the products was 1.1 s, for the labels 0.5 s, and for the prices 0.31 s. The participants fixated on trail mix (2.34 s), oat biscuits (1.79 s), and apple chips (1.75 s) the longest. For the labels, the ones of oat bar (1.04 s), trail mix (1.01 s), and Coke 1 (0.84 s) were fixated the longest, for the prices, the ones of apple chips (0.59 s), chocolate muesli bar (0.48 s), and Coke sugar-free (0.47 s). For details, please refer to Table A3. The products were observed significantly longer than the labels (*p* ≤ 0.001) and then the prices (*p* ≤ 0.001); further, the labels were observed significantly longer than the prices (*p* ≤ 0.001). In regards to a comparison of the different health value product groups, the AOIs of the healthier products were observed significantly longer than those of the unhealthier alternatives (*p* ≤ 0.01); their price signs and labels showed no significant differences. A more detailed comparison between the products with differing health values but with a comparable number of minutes (healthy products–low minutes vs. unhealthy products–low minutes and healthy products–high minutes vs. unhealthy products–high minutes) showed that the healthy products with the low minutes were observed significantly longer compared to their unhealthy alternatives (*p* ≤ 0.05). This was also true for the healthy products with high minutes (*p* ≤ 0.001); they were observed significantly longer and more intensely compared to their unhealthy alternatives.

#### 3.2.6. Sociodemographic Gaze Behavior

In regards to sociodemographic variables that might have influenced participants’ gaze behavior, we investigated that the younger half of the participants (= less than 26 years, mean age = 22.9 years) fixated the price signs earlier and faster (*p* ≤ 0.05) than the older half of the participants (older than 26, mean age = 30 years). Further, men fixated the price signs significantly less often than women (*p* ≤ 0.05). In regards to the movement behavior of the participants, we investigated differences between the group that reported higher activity levels (*n* = 58) and the group that reported less (*n* = 33). For the group that reported higher activity levels, the results showed a quicker response (time to first fixation) to the products as well as to the labels (products: *p* ≤ 0.05, labels: *p* ≤ 0.001), and a shorter fixation duration of the labels (*p* ≤ 0.05).

### 3.3. The Product Choice

The results of the selected products are shown in Table 3. In total, 162 products were chosen because multiple choices were possible, without limitation (similar to a real scenario). The most frequently chosen products were bananas (24×), water (14×), mate tea (13×), and trail mix (13×). All of these products were in the group with a perceived higher health value. We asked participants whether their choice corresponded to the choices they often made in everyday life. Here, 80.2% agreed, approximately 8% were unsure, and 12% disagreed. The participants were also asked whether the PACE labels influenced their purchase decision. A total of 12.1% agreed, 78% disagreed, and almost 10% were unsure. In contrast, when participants were asked whether the product price influenced their purchase decision, 50.5% agreed, almost 10% were unsure, and 38% disagreed. In regards to the two groups with differing physical activity, it can be seen that, in the group that was physically more active, 63.8% of the products purchased were from the group with healthier products, whereas in the group with the less physically active participants 75.8% of the products purchased were from the healthy product group.

### 3.4. Open Questions

With three open questions, we aimed to find out whether the participants recognized the PACE labels and what information they were looking for on the products. For the first question: “What did you look for when purchasing snacks?” we classified from the participants’ answers 206 content aspects into eight categories. Nearly 20% of the participants mentioned that they were looking for information about the product (e.g., origin, product appearance, etc.) or for price information. Moreover, 18% of the participants provided answers that we classified as product range (e.g., categories, variety, range arrangement, etc.), nearly 17% reported on their actual preferences and needs (e.g., food vs. drinks), and nearly 9% were looking for the health value of a product. Further, we classified a category dealing with answers concerning ingredients (8.25%), search criteria (5.34%), and the PACE label (2.91%).

For the second question: “Did you notice or remember anything special?” we classified 167 content aspects into six different categories, with half of the aspects being classified into the category product range (50.9%) (e.g., single products, product choice, etc.). Nearly 15% of the answers dealt with the product placement (e.g., shelf, setup, etc.), nearly 12% with the price and price comparisons, and nearly 9% with the health value of the products. Further, 8% indicated that they noticed the PACE label, whereas 6% mentioned that they did not notice or remember anything special.

For the third question: “What were you looking at or searching for?” we classified 190 content aspects, and identified two main categories, which were “snack motives” (67.37%) and “product features” (32.63%). To obtain a better understanding of what participants were specifically looking for, we also reported several subcategories of these two motives. The main “snack motives” were looking for healthy products and personal (taste) preferences (both 13.16%), followed by a small snack for in between (11.05%), drinks (9.47%), and sweets (8.97%). Two other motives were “snacks/foods that wake me up and give me energy” (6.84%) as well as “foodstuff that keeps me satiated for a long time” (4.74%). The category “product feature” highlighted which specific products from the product range the participants were looking for (13.16%), and what ingredients or special product characteristics were important to them (11.58%); moreover, some of them were looking for prices (5.79%) and for the PACE labels (2.11%).

## 4. Discussion

The purpose of this study was to obtain a better understanding of consumer perceptions of PACE labels, on a variety of real snack products, and the attention they received in a simulated food purchase setting. The special feature of this study was the application of a head-mounted eye-tracker system that allowed the participants to move freely, pick products off a shelf, and inspect the packages on their own initiatives, instead of being provided with images on a computer screen.

In regards to the perception of PACE labels, our eye-tracking data showed that the PACE labels in this study were looked at longer and more intensely compared to the price labels of the products. This is in line with other studies that showed that participants needed more time to perceive the labels, because processing the pricing information is easier than processing the more complex product information label, especially because price information is familiar to people and, therefore, does not attract much attention, whereas PACE labels are something individuals do not know about [27,28].

We observed large differences in terms of the visual attention for the PACE labels on different products, which is reflected in a high standard deviation of all three metrics (TFD: mean: 0.55 s, SD: 0.57; TTF: mean: 22.46 s, SD: 18.9; FC: mean: 1.39 s, SD: 0.73). This is in line with another study that used real products and a head-mounted eye-tracker as well and found that the visual attention for labels varied between different label claims on different product categories [29]. One explanation for the differences in this study might be the wide range of offered products. Despite the fact that we included products based on market availability, as well as health, we did not control for individual familiarity of all products (e.g., the organic products). As consumers tend to look longer at novel items [30,31], the novelty of some of the products and of the label (at individual respondent levels) per se could have led to the very different observation patterns. However, the sample size of 91 participants with a product choice of 162 was high, allowing for enough statistical variety among the participants and products to allow answering the main research questions, in regards to effectiveness and awareness of the PACE labels. 

In regards to the health value of the products, the healthier products were observed significantly longer than those of the unhealthier alternatives; however, there was no significant difference between the fixations of their labels. One explanation for this might be that we did not specifically ask participants to choose healthy products, so they were not specifically looking for or using the support of a label to classify the products. Since we did not ask the participants what to look for specifically, the variety between the possible individual “search goals” might have been very high. This would have been supported by the fact that we identified seven different snack motives (in total 67.37% of all answers) and four different product features/characteristics when the participants were asked what they were looking at or searching for. Furthermore, it seems that our subjects already had high levels of health interests. The participants were all university students, a segment of the population that demonstrates a relatively high interest in nutrition and nutrition labels [32]. Moreover, according to the direct ratings, participants exercised on a relatively regular basis, and most had a normal BMI. This might support the assumption of a rather health conscious sample.

Moreover, the general choices participants made were rather healthy, although no specific “health” goal was set for the participants. Bananas were the most commonly chosen products, and they had one of the slowest times until entering the respective label AOI. One explanation for this might be that the participants were specifically looking for a specific snack or fruits and, thereby, were fixated on the banana. Proportionately late first fixations often indicate top–down perceptions, since subjects deliberately scan the test setup for certain pieces of information [23]. It might have been the case here that consumer specific goals, e.g., healthiness or habits, influenced participant gaze behaviors to that point [33]. This is also supported by the direct reporting that indicated a search for “healthy products” (see above).

The group of participants that reported less physical activity looked later at the products and at the labels, but they looked more intensely at the labels. Additionally, this group purchased a larger percentage of products from the healthy product group. Higher fixation durations are associated with an increased probability of purchase, and because attention mediates the effect of nutrition labels on choice, products fixated most and longest have the highest likelihood of being chosen [11,34]. This could explain the higher percentage of healthy products bought by this group. However, contrary to our results, other studies showed that people trying to lose weight viewed calorie information longer because they were actively seeking for information [21]. Generally, it is known that more health-focused people are more willing to check labeling, because they are more interested in healthy behavior; however, this is not always effective in decreasing calorie information or leading to healthier choices [35,36]. One explanation for the present results could be that the used PACE labels were not directly perceived as a health-related label, but rather the movement instructions were in the foreground and were therefore not so much of interest to the group of subjects who already moved a lot anyway. The less active group however seemed to have used the labels, which supports the idea that labels should be easily and quickly understandable, providing information in an intuitive and highly visible way. Further, PACE labels may have unveiled already existing knowledge about healthy food choices, and most likely were included in the purchase choice, as the PACE labels were visually more important than the price, typically a very important product attribute. Hence, we conclude that the labels may be effective enough to “re-prime” (prime already existing information) and, in combination with a healthy product offer, lead to a more healthy product choice.

In regards to the general perception of the PACE label, 12% of the participants agreed that the PACE labels influenced their purchase decision, 8% indicated that they noticed the PACE label, and around 3% actively mentioned the use of the PACE labels when asked what they were looking for. At the same time, the average participant viewed the PACE label at least once on 22.9% of the products. Even though our labels were unknown to the participants, which could have increased the interest or attention to them, it seems that they did not attract a lot of direct attention. This is in line with other studies that showed that nutrition labels were not the most intensively viewed product information [31]. PACE labels, in a study by [21], achieved similar results; the average participant viewed the label on 17% of 64 nutrition fact labels. Other studies showed that nutrition labels were more likely to be used and viewed if health was generally important to the consumer or if consumers had special health goals already in mind [31,37]. If this holds true, it indicates that, due to the rather health conscious sample we had, the number of people who perceived or used such a label in real life could be much smaller. Nevertheless, the eye-tracking data suggest that the use of the labels may have been more impactful than the price. Using this as a set reference, being a decisive criteria, in turn, the PACE label and its effect may be very impactful, especially for people not very involved in healthier product choices.

Although only a very small proportion of the subjects mentioned that they recognized the PACE labels, and other participants reported that they did not notice the labels until they were explicitly asked about them in the ensuing questionnaire, it might have also been the case that the exposure to the PACE labels might have made people aware, priming them to choose healthier (although with our data, we cannot clarify this to the latest). This may have primed respondents to be healthy and push them toward healthy products, e.g., something that did not require much exercise to compensate for, such as the bananas, which were the only real fruit and scored rather low on the minutes, as well as water and mate tea, the second and third most purchased products. Nevertheless, the eye-tracking system is an indirect measurement system of consumers’ awareness; hence, despite the fact that we cannot probe “no-social-bias”, it is certainly less impactful than in sole questionnaire studies. Further studies could include a control group that is not exposed to the labels to clarify these aspects.

## 5. Strengths and Limitations

This study has a number of strengths: it stands out with a range of 20 different snack products under simulated real-world purchase conditions, offering an equal share of healthy and not-so-healthy snacks. The real-supermarket lab brought elements of realism into the study that were usually missing. Comparative studies were carried out under controlled conditions on a PC monitor, with a small number of products (usually 4–8 products; e.g., biscuits, yoghurts, soft drinks). This close-to-realistic environment with three-dimensional products and the head-mounted eye-tracker allowed us to examine human decision patterns in a natural environment, free from any constraints.

However, in regards to limitations, it must be mentioned that the set-up might have been affected by biases in regards to product placing, familiarity, and social desirability; however, we specifically used the indirect measurement of the eye-tracking system to combat social-response bias.

In regards to product placement, the product offers were not varied within subjects, which would have allowed controlling for patterns in gaze behavior e.g., people fixate from left to right and from top to bottom. This might have affected the product metrics to some point, because the products were observed longer and faster than the labels. Our placement was chosen to present the products as clearly as possible and distinctly from one another, but also to show them as realistic as a snack shelf. We analyzed the product placement and found it to be stable in regards to the awareness of the label and product category. The perception of the labels seems to not be (or only to a limited degree) influenced by this gaze patterns. As described, what was more important was the base level of information and physical activity in this regard. Further, we did not include a familiarity rating of the products at the individual respondent levels. Such a scale could enrich future studies in order to surely allocate the effect of familiarity in response to awareness. We also did not pretest the familiarity of the products we used and it might be that the participants looked longer at the rather novel or unfamiliar products, which makes it difficult to find out why products attracted participants’ attention. Overall, however, we are confident to pursue enough statistical variety through the chosen 162 products within our 91 respondents, in order to stabilize effects of familiarity or product placement, to answer the main research questions. Nevertheless, we would recommend randomizing the order and placing, if study budget can be allocated.

The use of three-dimensional products in a natural environment is challenging because they complicate a standardized preparation of the AOIs, as well as of the shelf. We chose the size of the PACE labels according to the size of the product packages, and tried to make them appear as realistic as they would in real-life. However, for the smaller products, such as the fruit bar or chocolate bar, we had to find a compromise with the size of the label that had to be realistic, but also large enough to be captured by the eye-tracker. Hence the labels were slightly too big for the small packages, but were still imaginable in a real-life scenario.

## 6. Conclusions

The present study was a first approach to examine PACE labels on various real snack products, with differing health values, in a close-to-realistic environment. The results indicate that PACE labels were perceived and actively remembered by the participants. They were looked at longer and more intensely compared to the price labels, but less often and intense compared to the product itself. However, as other product labels, they did not attract a lot of direct attention. Further, healthier products attracted more attention than unhealthier products. Moreover, the general product choices were rather healthy. This indicates the need to combine consumers’ processing information with the offers, which might have influenced their attention in regards to snack product choices, which is especially interesting for public health discussions.

Our results are interesting to the scientific and health communities, in regards to three key findings: (1) labels (as a means of health related information) can trigger more healthy buying behavior, especially for people not yet fully involved. (2) PACE labels, which reduce action-based information to simple, meaningful content (e.g., ‘running’ minutes) framed by a neutral tone (blue color) may be a meaningful pathway to combat non-communicable diseases and should be followed after. (3) We understand and discuss the effect of PACE labels in light of a re-priming (remember information, already known), and combine this with an action-based, easy to access hint (e.g., activity of xx minutes), as well as with a healthy product offer, which may be an important combination in order to improve public health.

Based on our results, one could additionally recommend being aware of different target groups that might use the label information in different ways; the capture of the labels through label size and product placement should be adjusted appropriately for people who are actively seeking assistance through labels. For this group, the PACE label may be the last tipping point to choose a healthier option, if healthy options are made available.

Our results also show that research in real-world settings is possible; therefore, we recommend continuing researching the influence of PACE labels on purchase behavior, with wider target groups, at best between-subjects, to better distinguish different experimental effects.

## Figures and Tables

**Table 1:** List of products with their calorie contents, equivalent values in jogging units, and the product price. The order of the products within the two groups (healthy vs. unhealthy) depicts the pairs described.

Products	Brand	Label	Calories per Product Unit (kcal)	Kcal per 100 g or mL	Kcal Equivalent in Jogging Minutes	Price in Euro
Healthy snacks						
Water, medium	Vio, medium	0 min	0	0	0	1
Orange juice	Hohes C	10 min	87.4	43.7	9.37	0.5
Mate tea	Club Mate	10 min	100	20	10.71	1.5
Fruit bar	Alnatura	15 min	135	338	14.15	0.5
Banana	-	15 min	115	89	12.32	0.5
Apple chips	Alnatura	25 min	213	356	22.82	1
Oat bar	Alnatura	30 min	276	406	29.58	1
Peanuts	Rapunzel	50 min	465.75	621	49.91	1
Trail mix	Seeberger	60 min	595	476	63.77	1.5
Oat biscuits	Bohlsener Mühle	60 min	596.25	477	63.9	1
Unhealthy snacks						
Coke, sugar-free	Coca Cola Zero	0 min	0	0	0	1
Fruit juice drink	Capri Sun	10 min	80	40	8.57	0.5
Energy drink	Red Bull	15 min	115	46	12.3	1.5
Chocolate muesli bar	Corny	15 min	114	455	12.21	0.5
Chocolate bar	Kinder Country	15 min	132	561	14.14	0.5
Coke	Coca Cola	25 min	210	42	22.5	1
Nut–nougat–crème snack	Nutella To Go	30 min	266	511	28.51	1
Potato chips	Funny-Frisch	30 min	265	530	28.4	1
Chocolate	Milka	60 min	530	530	56.8	1
Chocolate biscuits	Leibniz	60 min	610	488	63.89	1

**Table 2:** Sociodemographic characteristics of the sample (*N* = 91).

Characteristic	Description	Frequency	Percentage
Sex	Female	52	57.1%
	Male	39	42.9%
Age	18–24 years	37	40.7%
	25–30 years	39	42.8%
	31–36 years	8	8.8%
	37 years and older	7	7.7%
Educational Level	Without educational certificate	0	0
	Certificate of secondary education	0	0
	General certificate of secondary education	3	3.3%
	General qualification for university entrance	58	63.7%
	University degree	30	33.0%
Household size (*n* = 90)	Alone	21	23.1%
	With 1 other	28	30.8%
	With 2 others	21	23.1%
	With 3 others	7	7.7%
	With 4 others	2	2.2%
	With 5 others or more	11	12.1%
Employment	Full-time employment	1	1.1%
	Part-time employment	8	8.8%
	Student with part-time job	43	47.3%
	Student without part-time job	36	39.6%
	In apprenticeship	3	3.3%
Income	<EUR 600	21	23.1%
	EUR 600–899	36	39.6%
	EUR 900–1199	22	24.2%
	EUR 1200–1499	7	7.7%
	EUR 1500–1799	1	1.1%
	>EUR 1800	4	4.4%
Easy physical activity (=non sweating to slightly sweating) (*n* = 79)	Never	1	1.1%
	<1 h/week	8	8.7%
	1–2 h/week	9	9.8%
	>2 h/week	61	66.3%
Medium physical activity (slightly sweating) (*n* = 81)	Never	4	4.3%
	<1 h/week	21	22.8%
	1–2 h/week	23	25.0%
	>2 h/week	33	35.9%
Strong physical activity (heavily sweating) (*n* = 81)	Never	16	17.4%
	<1 h/week	15	16.3%
	1–2 h/week	20	21.7%
	>2 h/week	30	32.6%

**Table 3:** Frequency and percentage of the chosen products.

Product	Frequency	Percentage
**Banana**	24	26.4%
**Water, medium**	14	15.4%
**Mate tea**	13	14.3%
**Trail mix**	13	14.3%
**Oat bar**	11	12.1%
Chocolate bar	9	9.9%
**Peanuts**	8	8.8%
Chocolate	8	8.8%
**Fruit bar**	8	8.8%
Coke	7	7.7%
**Apple chips**	6	6.6%
Fruit juice drink	6	6.6%
Chocolate biscuits	6	6.6%
*No product*	6	6.6%
**Oat biscuits**	5	5.5%
**Orange juice**	5	5.5%
Nut–nougat–crème snack	5	5.5%
Potato chips	4	4.4%
Coke sugar-free	2	2.2%
Chocolate muesli bar	2	2.2%
Energy drink	0	0.0%

Note: multiple selections were possible. Bold letters represent the healthy products, normal letters the unhealthy one. The no-buy option is printed in italic letters.

## Data Availability

The data presented in this study are available upon request from the corresponding author.

## Appendix A

**Table A1:** Time to first fixation (TTF) (in seconds) and standard deviation (SD) of the products, the PACE label and the price in front of every product. Within the table, the data are sorted based on the minutes that were declared on the PACE label of the product.

AOI	Minutes on PACE Label	Time to First Fixation Product	SD	Time to First Fixation Label	SD	Time to First Fixation Price	SD
Coke sugar-free 1	0	13.63	17.21	18.87	14.76	49.53	46.41
Coke sugar-free 2	0	11.47	13.67	17.20	19.37	49.53	46.41
Water, medium 1	0	10.82	12.77	21.61	24.37	10.95	5.96
Water, medium 2	0	14.85	18.98	31.64	28.63	10.95	5.96
Mate tea	10	9.44	9.26	15.98	12.97	30.17	17.79
Orange juice 1	10	12.67	12.33	34.11	24.25	19.72	22.43
Orange juice 2	10	8.83	11.68	15.81	16.80	19.72	22.43
Fruit juice drink 1	10	5.82	7.55	10.17	11.48	6.65	5.18
Fruit juice drink 2	10	9.10	12.02	16.51	18.13	6.65	5.18
Fruit bar	15	16.37	14.18	26.65	23.06	11.50	4.13
Energy drink	15	11.92	13.00	13.72	14.39	9.11	9.37
Chocolate muesli bar	15	14.33	10.93	24.18	18.86	24.31	16.09
Banana 1	15	16.06	17.12	26.94	20.95	28.96	14.81
Banana 2	15	16.55	14.33	24.00	18.04	28.96	14.81
Chocolate bar	15	18.52	15.14	31.62	18.80	31.75	21.04
Apple chips	25	7.04	6.65	27.27	22.15	20.93	16.50
Coke 1	25	9.52	12.42	16.53	17.67	18.83	9.60
Coke 2	25	9.12	13.37	11.35	10.11	18.83	9.60
Potato chips	30	8.77	12.35	25.04	23.27	23.51	18.09
Nut-nougat-crème snack 1	30	14.13	12.82	24.36	17.62	19.34	14.80
Nut-nougat-crème snack 2	30	16.54	14.44	30.44	19.62	19.34	14.80
Oat bar	30	12.20	10.33	19.48	11.80	23.07	9.55
Peanuts	50	11.46	10.15	18.50	15.03	25.65	13.01
Trail mix	60	8.33	10.08	24.63	23.37	24.18	15.32
Oat biscuits	60	9.17	10.34	23.89	17.54	28.57	19.23
Chocolate	60	14.85	18.98	31.64	28.63	10.95	5.96
Chocolate biscuits	60	16.00	18.40	30.67	28.00	25.84	1.55
Average	-	11.99	12.76	22.47	18.98	22.37	14.90

Note: all products that were placed side-by-side received an individual AOI. Therefore, some products, such as the soft drinks, had two AOIs for the label. They are referred to as product 1 (the left product on the shelf) and product 2 (the right product on the shelf). The price label was placed between these two products, and similar products thus only had one price label. The TTF of the prices was therefore the same for product 1 and product 2.

**Table A2:** Number of fixations (FC) and standard deviation (SD) that the participants gave to the PACE labels, the products, and their prices. Within the table, the data are sorted based on the minutes that were declared on the PACE label of the product.

AOI	Minutes on PACE Label	Fixation Count Product	SD	Fixation Count Label	SD	Fixation Count Price	SD
Coke sugar-free 1	0	2.09	1.33	1.20	0.42	1.00	0.00
Coke sugar-free 2	0	2.30	2.09	1.19	0.68	1.00	0.00
Water, medium 1	0	2.87	2.19	1.50	1.10	1.17	0.41
Water, medium 2	0	2.67	2.15	1.71	0.99	1.17	0.41
Mate tea	10	3.96	3.18	1.21	0.41	1.00	0.00
Orange juice 1	10	1.98	1.58	1.07	0.27	1.13	0.35
Orange juice 2	10	2.58	1.53	1.26	0.56	1.13	0.35
Fruit juice drink 1	10	2.94	1.96	1.25	0.65	1.00	0.00
Fruit juice drink 2	10	3.07	1.84	1.14	0.48	1.00	0.00
Fruit bar	15	3.40	3.09	1.24	0.54	1.00	0.00
Energy drink	15	2.58	1.94	1.81	1.33	1.00	0.00
Chocolate muesli bar	15	2.64	2.85	1.17	0.38	1.00	0.00
Banana 1	15	3.07	1.84	1.45	0.96	1.20	0.45
Banana 2	15	3.51	3.02	1.60	1.13	1.20	0.45
Chocolate bar	15	2.76	2.12	1.80	0.89	1.25	0.50
Apple chips	25	5.54	4.80	1.27	0.59	1.50	0.55
Coke 1	25	2.84	2.00	1.69	1.14	1.00	0.00
Coke 2	25	2.53	1.63	1.00	0.00	1.00	0.00
Potato chips	30	4.66	3.67	1.38	0.77	1.14	0.38
Nut–nougat–crème snack 1	30	2.38	1.69	1.27	1.00	1.00	0.00
Nut–nougat–crème snack	30	2.26	1.76	1.39	0.61	1.00	0.00
Oat bar	30	4.04	3.50	1.70	1.36	1.00	0.00
Peanuts	50	3.98	3.11	1.09	0.30	1.00	0.00
Trail mix	60	6.66	5.58	1.88	1.20	1.17	0.41
Oat biscuits	60	5.10	3.94	1.48	0.92	1.11	0.33
Chocolate	60	3.35	2.21	1.29	0.59	1.30	0.67
Chocolate biscuits	60	3.95	2.65	1.50	0.65	1.00	0.00
Average	-	3.32	2.60	1.39	0.74	1.09	0.20

Note: all products that were placed side-by-side received an individual AOI. Therefore, some products, such as the soft drinks, had two AOIs for the label. They are referred to as product 1 (the left product on the shelf) and product 2 (the right product on the shelf). The price label was placed between these two products, and similar products thus only had one price label. The FC of the prices was therefore the same for product 1 and product 2.

**Table A3:** Total fixation duration (TFD) and standard deviation (SD) of the products, the PACE labels and the price signs (in seconds). Within the table, the data are sorted based on the minutes that were declared on the PACE label of the product.

AOI	Minutes on PACE Label	Total Fixation Duration Product	SD	Total Fixation Duration Label	SD	Total Fixation Duration Price	SD
Coke sugar-free 1	0	0.69	0.65	0.66	0.86	0.47	0.03
Coke sugar-free 2	0	0.88	0.91	0.47	0.52	0.47	0.03
Water, medium 1	0	0.82	0.80	0.74	1.12	0.29	0.16
Water, medium 2	0	1.01	1.08	0.75	0.59	0.29	0.16
Mate tea	10	1.29	1.27	0.39	0.35	0.35	0.13
Orange juice 1	10	0.77	0.92	0.35	0.29	0.39	0.29
Orange juice 2	10	0.84	0.67	0.46	0.44	0.39	0.29
Fruit juice drink 1	10	0.87	0.75	0.54	0.66	0.23	0.15
Fruit juice drink 2	10	0.89	0.62	0.42	0.29	0.23	0.15
Fruit bar	15	1.13	1.39	0.55	0.68	0.23	0.01
Energy drink	15	0.77	0.78	0.55	0.68	0.20	0.03
Chocolate muesli bar	15	0.84	1.02	0.45	0.32	0.48	0.27
Banana 1	15	1.02	1.28	0.34	0.66	0.24	0.07
Banana 2	15	1.27	1.54	0.66	0.56	0.24	0.07
Chocolate bar	15	0.90	0.78	0.48	0.40	0.24	0.07
Apple chips	25	1.75	1.73	0.45	0.35	0.59	0.29
Coke 1	25	0.87	0.87	0.84	0.84	0.30	0.13
Coke 2	25	0.67	0.60	0.33	0.19	0.30	0.13
Potato chips	30	1.42	2.60	0.50	0.65	0.36	0.31
Nut–nougat–crème snack 1	30	0.72	0.71	0.51	0.60	0.20	0.13
Nut–nougat–crème snack 2	30	0.67	0.73	0.45	0.41	0.20	0.13
Oat bar	30	1.71	2.11	1.04	1.27	0.17	0.09
Peanuts	50	1.39	1.28	0.28	0.11	0.21	0.03
Trail mix	60	2.34	2.48	1.01	0.90	0.29	0.08
Oat biscuits	60	1.79	1.76	0.63	0.64	0.46	0.36
Chocolate	60	1.05	0.85	0.61	0.79	0.44	0.21
Chocolate biscuits	60	1.25	1.22	0.48	0.29	0.22	0.06
Average	-	1.10	1.16	0.55	0.56	0.31	0.15

Note: all products that were placed side-by-side received an individual AOI. Therefore, some products, such as the soft drinks, had two AOIs for the label. They are referred to as product 1 (the left product on the shelf) and product 2 (the right product on the shelf). The price label was placed between these two products, and similar products thus only had one price label. The TFD of the prices was therefore the same for product 1 and product 2.
